# Defibrillation Testing of the Implantable Cardioverter Defibrillator: When, How, and by Whom?

**Published:** 2007-08-01

**Authors:** Luis A Pires

**Affiliations:** Heart Rhythm Center, Division of Cardiovascular Medicine, Department of Medicine, StJohn Hospital and Medical Center, Detroit, MI

**Keywords:** defibrillation, ICD, testing, ventricular fibrillation

## Abstract

The implantable cardioverter-defibrillator (ICD) has become an integral part of treatment for a variety of patients with symptomatic, or at risk for, ventricular tachyarrhythmias. The ICD's effectiveness is attributed to its ability to promptly detect and terminate ventricular tachycardia (VT) and fibrillation (VF). The clinical trials that established the positive role of ICD therapy were based on patients who underwent some form of defibrillation testing at the time of implantation. Therefore, since its advent, intraoperative defibrillation testing of the ICD to assure reliable detection and termination of VT/VF has been a standard practice. But because of advances in defibrillator and lead technology, which now facilitates successful device implantation (i.e., low defibrillation energy requirement to allow for an adequate programmed safety margin) in the majority of patients, the necessity of defibrillation testing has been called into attention. Despite substantial progress, it is not altogether clear whether a wholesale abandonment of intraoperative ICD testing is appropriate at this point. We review pertinent data regarding pros and cons of ICD testing and offer a suggestion as to when, how, and who should test ICDs.

Several clinical trials over the past decade have established the efficacy and benefit of the implantable cardioverter-defibrillator (ICD) in patients with documented [1], as well as those at increased risk of developing [2-4], sustained ventricular tachyarrhythmias. As a result ICD therapy has become standard therapy for a variety of patient groups [5]. Through its ability to detect and (promptly) terminate >95% of spontaneous episodes of ventricular tachycardia (VT) and ventricular fibrillation (VF), ICD therapy has been shown to reduce the risk of sudden death [6] and overall mortality [1-4]. Such observations were based on patients who underwent some form of intraoperative testing [1-4]. Therefore, ICD testing at the time of device implantation to confirm proper detection and successful termination of induced VT/VF has been the standard of care for several years [7-11]. The amount of energy (Joules) needed to terminate VF is used to establish a given patient's defibrillation energy requirement (DER).

Advances in defibrillator technology, most notably the use of biphasic shock waveforms [12-15], now facilitate successful device implantation (i.e., DER that is at least 10 J lower than the device's maximum output) in the majority of cases [16-19]. As a result, some investigators [20] have recently questioned the necessity of ICD testing altogether, noting that, among other considerations, forfeiting ICD testing might lead to an increase in the use of ICDs by allowing non-electrophysiologists with reduced training requirements [21], who may also be uncomfortable with ICD testing, to implant devices. Despite substantial improvement in device and lead technologies and the (probable) necessity to expand device therapy to a greater number of patients, current data do not support wholesale abandonment of ICD testing [19,22,23].

## When To Test

Device testing at the time of implantation has been a mainstay of such therapy since the advent of the ICD. Unless there are specific contraindications to testing (Table 1), VF should be induced to ensure that the ICD can reliably sense, detect and terminate the arrhythmia with an adequate shock energy (see below). Some of the contraindications are absolute and unavoidable (e.g., known cavity thrombus), but others can be overcome (such as assuring the presence of anesthesia staff to facilitate testing). Among our cohort of 835 consecutive patients, testing was not performed in 203 (24%), of which ~70% were due to the presence of cavity thrombi or inadequate anticoagulation and intraoperative hypotension [19]. Other investigators have reported similar results [22], and in general in up to a third of patients, testing may have to be postponed. Recently some have postponed intraoperative testing in patients undergoing cardiac resynchronization therapy (CRT) devices for fear of coronary sinus lead dislodgment [24], but we have not found this to be a significant problem among a cohort of >500 patients (unpublished observation). Patients who do not undergo intraoperative testing should be reassessed in the near future (usually 4 to 6 weeks) and testing reconsidered once the contraindications have been resolved.

With rare exceptions noninvasive postoperative testing of ICDs, either predischarge or several weeks post-implantation, may not be required [25-29] unless there are specific circumstances in which an increase in DER is expected, most notably the addition of amiodarone therapy. Because amiodarone increases DER by as much as 50% [30-33], and can also result in significant slowing of VT rate and, therefore, need for ICD reprogramming, device retesting should be strongly considered in patients receiving it. In cases where intraoperative DFT is ≤15 J, however, ICD retesting may not be required since the drug's impact on defibrillation energy safety margin is small with modern ICDs [33]. It should be noted, however, that this may not apply to patients with reduced (<20%) left ventricular ejection fraction (LVEF) since those patients were excluded from the study [33], a substantial proportion of ICD recipients in general. Although several variables, including LVEF, have been shown to influence intraoperative DER [23], there are no specific discriminators that can adequately predict which patients are expected to have high or low DER. Foregoing testing in patients with low LVEF (at our center we use an arbitrary cutoff value of <10%) may have the unintended consequence of possible device failure among patients at potentially greater risk of developing spontaneous VT/VF and for whom repeated unsuccessful shocks are more likely to lead to post-shock pump failure [34-37].

As for testing at the time of ICD generator replacements, in addition to confirming proper integrity of the chronic leads [38], we recommend defibrillation testing unless there had been appropriate therapies against VF in the near past and no evidence of substantial progression in the patient's cardiac disease and/or the addition of drugs (e.g., amiodarone) that may adversely raise DER. In clinically stable patients whose devices are not being replaced and who have not received any successful therapies against VF for a period of time, defibrillation testing might be reasonable albeit there is limited data regarding its usefulness [28].

## How To Test and the Impact of Testing

Prior to defibrillation testing, adequate sensing (>5 mV) and pacing (<1 V) parameters and electrical integrity (connections) between the leads and pulse generator should be determined and recorded. And assuming no contraindications (Table 1), with the patient comfortably sedated, VF is induced (through a variety of methods) with the following goals: (1) assure proper sensing and detection (and in case of first-shock failures, redetection) of VF; and (2) establish DER, based on which the device is then optimally programmed.

There are two broad categories of defibrillation testing: defibrillation threshold (DFT) and defibrillation safety margin (DSM). The DFT, defined as the lowest amount of energy capable of terminating an episode of induced VF, is most commonly determined through a step-down method (i.e., successive lowering of shock strength). Because success of defibrillation is probabilistic, a true DFT cannot be established with certainty. For this reason plus the potential risk of repeated inductions of VF and defibrillations [36-37], determining a DFT is now rarely used clinically except for research purposes [28,39]. In clinical setting, the goal of ICD testing is to determine an energy level that has a reasonable chance of success against spontaneous VT/VF events. This can be established by confirming a simple DSM, defined as an energy level capable of terminating one or more (generally two) episodes of induced VF and low enough to be at least ≥10 J less than the device's maximum output [16-18]. This 10 J "safety margin", achievable in nearly all patients with modern ICDs [16-18,28,39,40], has long been a common practice [41] because patients with elevated DER (and monophasic devices) were thought to have a higher mortality rate [42,44]. With modern, biphasic devices, elevated DER has not been shown to adversely impact patient survival [45,46]. Nonetheless, though the required magnitude of the safety margin energy is uncertain and debated [47], its establishment and incorporation in ICD programming allows for uncertainties in the DER requirement over time due to factors such as the addition of antiarrhythmic drugs, progression of cardiac disease, acute coronary events, and electrolyte changes.

**Figure 1:**
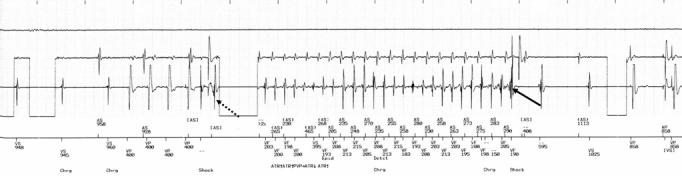
From top to bottom: atrial electrograms, ventricular electrograms, and marker channel. AS and VS indicates, respectively, atrial sensing and ventricular sensing. Shows an episode of induced ventricular fibrillation (dotted arrow) followed by a single successful 11 J shock (arrow) delivered by a 31 J output device.

**Figure 2:**
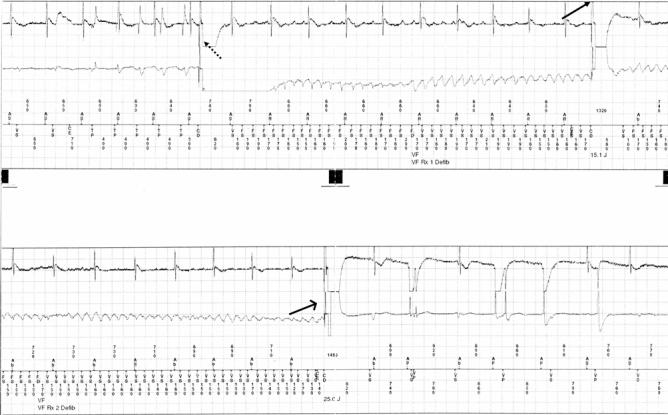
From top to bottom: atrial electrograms, ventricular electrograms, and marker channel. The tracing is continuous (top to bottom panel). Shows an episode of induced ventricular fibrillation (dotted arrow) followed by a failed 15 J shock (arrow), followed by a second successful 25 J shock (open arrow) delivered by a 35 J output device. AS and VS indicates, respectively, atrial and ventricular sensing.

The confirmation of a single defibrillation success at 10-15 J (with devices with maximum outputs of 30-35 J) predict successes with stronger (20-30 J) shocks with a nearly 100% accuracy [16-19]. Repeat testing at the same or lower energy levels does not seem to be clinically important and in our laboratory we follow a simple testing protocol: we test once at 11 or 15 J depending on the device's maximum output (Figure 1); and if the first shock fails, a second shock of 10 J greater (21 or 25 J) is delivered (Figure 2). If the second shock fails, after reconfirming proper lead positions, we would then change shock polarity and tilt (StJude devices) and repeat the process. With an adequate programmed safety margin (generally ≥10 J), the first shock success rates against spontaneous VT/VF episodes range from 83% to 93% [16-19,22], suggesting that defibrillation failures for spontaneous VT/VF may be due to factors (e.g., heart disease progression) not present at the time of intraoperative ICD testing. Therefore, it is not surprising that rigorous intraoperative testing may not necessarily translate into a fail-safe device therapy [19,34,35]

Despite a surge in the use of ICDs, there has been limited data on the impact of ICD testing on patient outcome, in terms of both defibrillation success against spontaneous VT/VF events and patient survival [19,22]. When comparing the outcome of ICD recipients who underwent DFT, DSM and no testing at the time of implantation, we recently reported similar success of ICD therapies and sudden-death-free survival among the three groups, however, overall survival was significantly worse in the no-testing group but similar in the DFT and DSM groups [19]. The comparable survival rates in the DFT and DSM tested groups are important given that for many years now DSM testing has replaced DFT testing in clinical settings. The higher mortality in the no-testing group probably reflected the inclusion of sicker patients, and not the result of device therapy failure [19]. But since we could not confirm the specific causes of death in all cases, in the absence of prospectively obtained corroborating data we recommended against abandoning testing altogether, in line with other investigators [22].

The ICD's primary function is to abort would-be fatal tachyarrhythmic events. Despite its phenomenal efficacy in terminating spontaneous VT/VF events, the ICDs protection against sudden death is not absolute [19,34,35,48]. With rare exceptions [49], device malfunctions very infrequently account for sudden death in ICD recipients [34,35]. Often such deaths are the result of an acute cardiac (e.g., acute myocardial infarction) or non-cardiac (e.g, vascular rupture) process in the setting of a normally functioning ICD - factors that cannot be anticipated or tested for at the time of implant [34,35].

## Who Should Test

ICD testing has always been the domain of cardiac electrophysiologists, beginning with  epicardial devices and later with the advent of first-generation transvenous devices, both of which were implanted by cardiac surgeons. In light of recent proposal sanctioning alternative training pathways in device implantation [21], and heavy industry promotion, device testing (usually DSM) is now also performed by non-electrophysiologists. Whereas operator volume may impact patient outcome [50], there exists no data on the safety and outcome of patients treated by non-electrophysiologists, making specific recommendations as who should test ICDs problematic. Nonetheless it should be noted that non-electrophysiologists are not expected to participate in implantation of devices in patients with documented VT/VF (i.e., secondary prevention) who may be at greater future risk and, therefore, for whom careful testing is important to assure proper device function. Moreover, non-electrophysiologists, who may have suboptimal training in defibrillation, must be fully aware of patient and device specific factors that influence defibrillation success and proper troubleshooting methods to insure successful implantation in each case [40]. When there is sufficient doubt about the outcome, testing should be left to an experienced electrophysiologist.

## Conclusions

**Figure 3:**
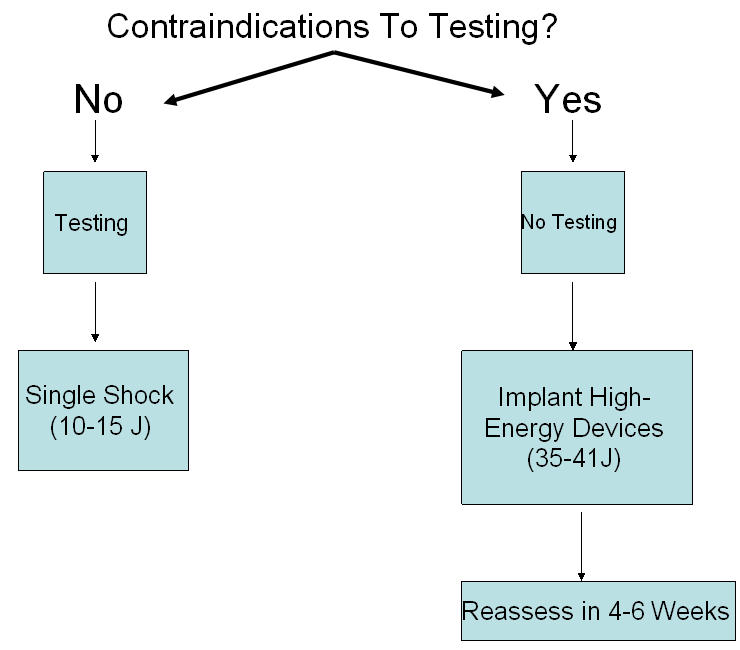
A suggested approach to intraoperative ICD testing. See text for details.

With current ICDs, successful device implantation (i.e., a DER energy allowing for a ≥10 J programming safety margin), determined through a single test and one episode of induced VF, can be expected in nearly all patients. Since such minimal testing rarely results in adverse events and there are no prospective data on the outcome of ICD recipients whose devices are not tested intraoperatively, we feel that, in the absence of contraindications, a minimum of testing is still appropriate (Figure 3). Certainly we do not believe testing should be abandoned to simply facilitate more device implantations by non-electrophysiologists who may be in some cases uncomfortable with defibrillation testing.

## Figures and Tables

**Table 1:**
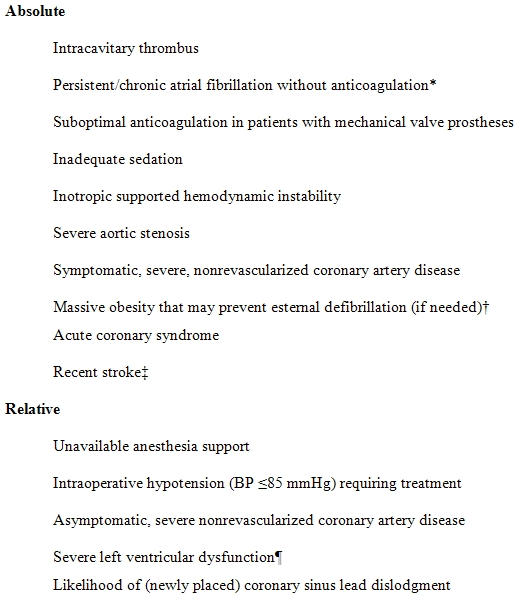
Contraindications to Intraoperative ICD Testing

*Unless there is no thrombus on transesophageal echocardiography. †Especially in patients with anticipated high defibrillation energy requirement (increasing the likelihood of needed external shock). ‡The best timing is uncertain, but we generally wait 4 to 6 weeks. ¶ We use a cutoff of ≤10% ejection fraction; others have used <20% [33].
